# Increasing Prevalence of Long‐Term Antidepressant Use in Australia: A Retrospective Observational Study

**DOI:** 10.1002/pds.70267

**Published:** 2025-11-15

**Authors:** R. A. D. L. M. K. Ranwala, Elizabeth E. Roughead, Jean‐Pierre Calabretto, Andre Q. Andrade

**Affiliations:** ^1^ Quality Use of Medicines and Pharmacy Research Centre, UniSA Clinical and Health Sciences University of South Australia Adelaide South Australia Australia; ^2^ School of Pharmacy and Medical Sciences University of South Australia Adelaide South Australia Australia

**Keywords:** antidepressant prescribing trends, deprescribing, long‐term antidepressant use, medication safety, overprescribing

## Abstract

**Background:**

Long‐term antidepressant use may reduce the risk–benefit profile due to the increased likelihood of withdrawal symptoms and higher incidence of side effects. This epidemiological study investigates historical trends in long‐term antidepressant use, which was defined as maintaining continuous antidepressant use for at least 365 days, allowing for gaps in dispensing of up to 60 days in the Australian community from 2014 to 2023.

**Method:**

A retrospective analysis was conducted using a 10% sample of data from the Australian Pharmaceutical Benefits Scheme (PBS), including patients aged over 10 years who had been dispensed a PBS‐listed antidepressant between January 2014 and December 2023.

**Results:**

From 2014 to 2023, the prevalence of long‐term antidepressant use increased from 66.1 to 84.6 per 1000 population. Age‐stratified analysis showed that the 10–24 age group had the highest relative increase in long‐term user prevalence (110%) and in the proportion of long‐term users (35%). The average duration of the treatment episode increased by 25% across all ages, with the 10–24 group showing the largest rise (56%). The percentage of long‐term users with apparent dose reductions showed minimal change over time.

**Conclusions:**

The study highlights a growing trend in long‐term antidepressant use across all age groups, particularly among those aged 10–24, warranting further investigation into the underlying factors. The extended treatment duration, coupled with limited medicine apparent dose reduction efforts, may suggest overprescription and underuse of deprescribing strategies. A more comprehensive mental health approach is needed, integrating effective deprescribing practices and emerging technological interventions.

**Summary**

- *Rising long‐term use (2014–2023)*: Long‐term antidepressant prevalence and average treatment duration increased steadily across all age groups.
- *Youth most affected*: The 10–24 age group showed the highest relative increase in long‐term use and treatment duration.
- *Limited apparent dose reduction*: No substantial change was observed in the proportion of users reducing antidepressant Defined Daily Dose.
- *Potential overprescribing*: Prolonged use without increased efforts to reduce medication strength suggests inadequate use of deprescribing practices.
- *Need for better deprescribing practices*: Results highlight the need for targeted deprescribing strategies, especially for youth, and greater integration of technology in mental health care.

## 1 Introduction

Antidepressants are widely prescribed psychotropic medications, primarily indicated for the management of moderate to severe depression [1]. Some antidepressants are also approved for managing anxiety [2] and panic disorders [3]. However, clinical practice shows that prescribing patterns extend beyond officially approved therapeutic uses, with notable instances of off‐label prescribing [4]. A study conducted in a community youth mental health service in Australia found that antidepressants and antipsychotics were mostly prescribed off‐label medicine in adolescents by public psychiatric staff [5]. Another study conducted using over 100 000 antidepressant prescriptions written by 150 physicians in Canada revealed that about 29% of all prescribed antidepressant prescriptions were off‐label indications, especially for insomnia and pain management [6].

Clinical guidelines recommend an antidepressant therapy duration of 6–12 months to achieve optimal therapeutic benefits in depression management, with extended treatment durations potentially required for high‐risk patients to minimise relapse risk due to the recurrent nature of depressive disorders [1, 7]. However, a growing trend of long‐term antidepressant use, defined as medication use over 1 year or longer, has been documented in multiple countries, including the United Kingdom, Italy and Sweden [8, 9, 10]. Further, a cross‐sectional analysis of antidepressant users in Australian general practices found that about one‐third continue long‐term use without clear medical indications or clinical justification [11].

Long‐term antidepressant users who discontinue their medication face a substantial risk of withdrawal symptoms, which can present with varying prevalence, severity and duration [12]. Severe withdrawal symptoms may be misinterpreted as disease relapse, potentially leading to unnecessary medication reinstatement and creating a cycle of continued use [13]. Further, the prolonged utilisation of antidepressants has raised significant clinical concerns regarding the balance between therapeutic benefits and potential adverse effects [14]. At the healthcare system level, prolonged and potentially unwanted antidepressant use may lead to increased pharmaceutical expenditure [15].

Multiple factors contribute to the continued use of antidepressants, including perspectives from both healthcare providers and patients. A systematic review of healthcare providers' perspectives on antidepressant discontinuation among long‐term users identified several key barriers [16]. These included concerns about relapse, beliefs in the long‐term effectiveness and safety of the medication and the perception that discontinuation is a time‐consuming and resource‐intensive process. Another study found that patients who undergo frequent medical reviews are more likely to experience medication adjustments, whereas those without regular follow‐ups tend to become long‐term users [17]. Regarding patient‐related factors, a systematic review on barriers and facilitators of long‐term antidepressant use conducted in 2019 found that patients were more likely to continue medication if they lacked coping strategies, faced unstable life circumstances, or did not experience significant adverse effects [18]. The review also reported that patients expected healthcare providers to initiate discussions about the discontinuation process, and patients expressed a willingness to follow their physicians' recommendations. Recent population‐based studies conducted in Sweden and Bologna, Italy, have identified additional factors associated with long‐term antidepressant use, including older age, female gender, urban residence and nursing home status [19], as well as antidepressant polytherapy and initiation of antidepressants by a hospital physician [10].

Despite the significance of long‐term antidepressant use, a notable knowledge gap exists in our understanding of long‐term antidepressant utilisation patterns within the Australian context. Specifically, research is limited regarding changes in prevalence over time and patient characteristics associated with long‐term antidepressant usage. Closing these knowledge gaps would be helpful for informing evidence‐based prescribing practices, guiding policy decisions and enhancing mental health care in Australia. The present study aimed to understand the trends in long‐term antidepressant use over the decade spanning 2014–2023, utilising data from the Pharmaceutical Benefits Scheme (PBS). We conducted an analysis of the incidence of antidepressant users across various age cohorts and examined the subsequent patterns of long‐term use within these demographic groups.

## 2 Methods

### 2.1 Study Design and Data Source

We conducted a descriptive observational retrospective analysis of antidepressant medication use between 2014 and 2023 using a 10% sample of the PBS dispensing claims data. The PBS data, maintained by Services Australia, contains prescription medicine claims for all Australian citizens and permanent residents. The PBS data excludes most of the medications dispensed to inpatients in public hospitals and private purchases. The de‐identified dataset includes individual information (gender, birth year) and dispensing details (prescription and dispensing dates, medicine Anatomical Therapeutic Chemical (ATC) code, quantity of supplies). Children under 10 years of age were excluded from the analysis as antidepressants are not generally indicated in this age group. Approval was obtained from the Department of Human Services External Request Evaluation Committee (EREC).

### 2.2 Medications of Interest

We selected antidepressant medications listed in the ATC Classification System by the World Health Organisation. Amitriptyline and Bupropion were excluded due to their common use for other indications, such as neuropathic pain management [20] and smoking cessation [21], respectively.

### 2.3 Data Extraction and Definitions

Data from the client and dispensing tables were merged using unique identifiers. Gender, year of birth, date of prescription, date of dispensing, ATC code, PBS code of the drug, strength and quantity of supplies were extracted. Given the lack of standardised definitions for antidepressant use in the literature, we employed the following specific criteria:

1. *Antidepressant user*: An individual who fills at least one antidepressant prescription per year.
2. *Long‐term antidepressant user*: An individual maintaining continuous antidepressant use for at least 365 days, allowing for gaps in dispensing of up to 60 days (approximately 2 months). Continuous use was considered interrupted when there was a gap of more than 60 days between medication dispensing events. Following such an interruption, any subsequent dispensing was considered the beginning of a new treatment episode. This methodology enabled researchers to identify multiple episodes of medication use within a single individual's treatment history.
3. *New user*: New users were defined as individuals who were dispensed an antidepressant for the first time in the data set or those who had previously used antidepressants but had not received any dispensing for over 365 days since their last recorded use, thereby restarting the medication.
4. *Apparent dose reduction attempt*: Due to the absence of dose information in the PBS sample dataset, apparent dose reduction attempts were identified using changes in total medication supplied expressed as Defined Daily Dose (DDD) units per dispensing as a proxy measure for dose changes. DDD units per dispensing were calculated as:

$$\begin{array}{c} \mathrm{DDD}\;\text{units}\;\mathrm{per}\;\text{dispensing}=\\ \left(\text{Strength in}\;\mathrm{mg}\times \text{Quantity dispensed}\right)/\mathrm{DDD}\;\text{value} \end{array}$$

An apparent dose reduction attempt was defined as a decrease in DDD units between consecutive dispensing of the same antidepressant within the same treatment episode, with no new antidepressant prescriptions or strength increases in other antidepressants within the past 6 months.

### 2.4 Statistical Analysis

The 10% PBS dataset we received from Services Australia contains a complete capture of medicines dispensed under the PBS from July 2012. We required a minimum of 1 year of dispensing history to identify long‐term antidepressant users. Considering this requirement, we selected the period from 2014 to 2023 for our main analysis. However, to identify long‐term users in 2014, we also examined dispensing records from 2013 to determine whether individuals met the study's definition of a ‘Long‐term Antidepressant user,’ as outlined above. We excluded records lacking birth year information.

The annual crude incidence and prevalence rates of antidepressant users, as well as the rate of long‐term use, were calculated per 1000 population using mid‐year Estimated Resident Population (ERP) data from the Australian Bureau of Statistics. Age and sex‐standardisation was not performed, as the objective was to quantify the actual population‐level healthcare burden and prescribing trends over time, and a recent study suggested that differences in healthcare utilisation rates calculated using ERP versus Medicare enrolment data were minimal at the national level [22].

We stratified the distribution of long‐term antidepressant users by gender and age category. Age was calculated based on the year of medicine dispensing and categories according to generations listed in the Australian Bureau of Statistics [23]. Age‐stratified subgroup analyses were conducted to explore variations in antidepressant use across different age categories. We analysed age‐specific incidence proportions, prevalence rates, long‐term prevalence and the proportion of long‐term users within each age category for both 2014 and 2023. We also examined the relative increase in long‐term antidepressant use between 2014 and 2023. Additionally, we conducted subgroup analyses by antidepressant class, using ATC subcategories, to examine prescribing patterns across different medication types and age groups. For all analyses, 95% confidence intervals were calculated and reported.

Further, we examined the duration of long‐term antidepressant use. However, consistent estimation using actual dispensing data was not feasible across the entire study period due to data limitations. Left censoring led to underestimation among patients who initiated treatment prior to 2014, as PBS records before April 2012 lacked comprehensive capture of under co‐payment prescriptions, making it difficult to determine true initiation dates. Right censoring also affected individuals who commenced treatment in later years (particularly 2022–2023), due to insufficient follow‐up time to observe treatment cessation. Therefore, the average treatment duration of antidepressant use was estimated using the prevalence‐incidence relationship equation [24]. This epidemiological principle proposed that, under steady‐state conditions, the mean duration of treatment is equal to the ratio of prevalence to incidence. Given that antidepressant user prevalence may vary over time, we used the DDD per 1000 population per day as a proxy for the point prevalence of antidepressant use [25]. Data management and statistical analyses were performed using Python version 3.1.12 and related libraries (Pandas, matplotlib and Seaborn).

### 2.5 Sensitivity Analysis

To determine an appropriate interval between prescription supply dates for defining continuous use, we conducted a sensitivity analysis. We varied the maximum allowable gap between supplies to 15, 30, 60 and 180 days. Our analysis showed consistent trends in long‐term antidepressant use across these different definitions (Supporting Information S1). Stockpiling of medications, especially toward the end of the year in anticipation of price increases, is common among long‐term users in Australia [26]. This behaviour can result in legitimate gaps in dispensing records that do not reflect true discontinuation. Moreover, in the Australian healthcare context, it is unlikely that a patient discontinuing long‐term medication would completely cease treatment, experience symptom recurrence, consult a general practitioner and initiate a new treatment episode, all within a short period of 15 or 30 days. Therefore, we selected a 60‐day grace period as a more appropriate threshold for identifying ongoing treatment.

## 3 Results

Our analysis of the PBS 10% sample data of antidepressant users aged 10 years and above during the study period (2014–2023) showed an upward trajectory in prevalence. It increased from 107.7 per 1000 population in 2014 to 130.6 in 2022, with a notable acceleration during the COVID‐19 pandemic period (2019–2022). By 2023, the prevalence rate slightly decreased to 128.8 per 1000 population (Table 1).

**TABLE 1:** Antidepressant user distribution from 2014 to 2023.

Year	Incident antidepressant users	Prevalent antidepressant users	Incident users percentage	Incident proportion per 1000 population (95% CI)	Antidepressant users' prevalence per 1000 population (95% CI)
2014	60 124	220 201	27.3 (27.1–27.5)	29.4 (29.2–29.6)	107.7 (107.3–108.2)
2015	61 441	229 009	26.8 (26.6–27.0)	29.6 (29.4–29.9)	110.5 (110.1–110.9)
2016	62 465	237 432	26.3 (26.1–26.5)	29.7 (29.4–29.9)	112.8 (112.4–113.2)
2017	63 452	245 127	25.9 (25.7–26.1)	29.6 (29.4–29.8)	114.4 (114.0–114.8)
2018	64 963	254 200	25.6 (25.4–25.7)	29.8 (29.6–30.0)	116.6 (116.2–117.1)
2019	66 644	264 029	25.2 (25.1–25.4)	30.1 (29.8–30.3)	119.1 (118.7–119.5)
2020	70 050	276 092	25.4 (25.2–25.5)	31.1 (30.9–31.4)	122.7 (122.3–123.2)
2021	73 045	291 106	25.1 (24.9–25.2)	32.4 (32.1–32.6)	129.0 (128.6–129.5)
2022	70 122	298 959	23.5 (23.3–23.6)	30.6 (30.4–30.9)	130.6 (130.2–131.1)
2023	69 251	303 104	22.9 (22.7–23.0)	29.4 (29.2–29.6)	128.8 (128.4–129.2)

Abbreviation: 95% CI, 95% confidence interval.

The annual incidence proportion of antidepressant users increased from 29.4 per 1000 population in 2014 to 32.4 in 2021, then decreased to 29.4 in 2023. The proportion of incident users relative to prevalent users gradually declined over time, with the lowest percentage observed in 2023 at 22.9%.

### 3.1 Long‐Term Antidepressant Use

**FIGURE 1:**
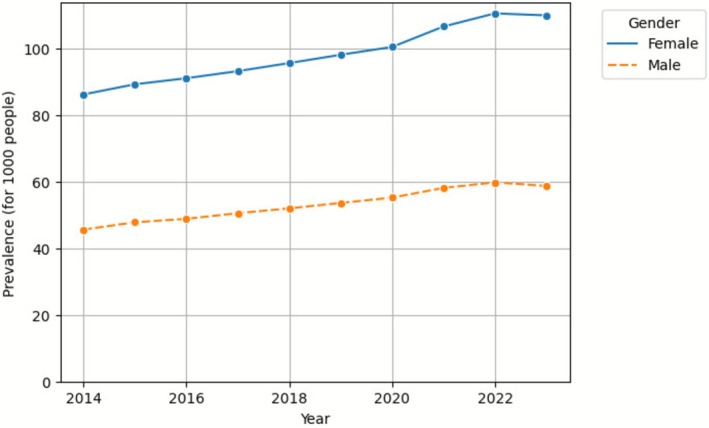
Prevalence of long‐term antidepressant use by gender.

We observed a continuous increase in long‐term use from 66.1 per 1000 population in 2014 to 85.6 in 2022 (Table 2). Gender‐stratified analysis revealed a consistently higher prevalence of long‐term use among female users throughout the study period (Figure 1).

**TABLE 2:** Long‐term antidepressant user distribution from 2014 to 2023 year.

Year	Long‐term antidepressant users	Long‐term users' percentage among all users	Long‐term users' prevalence per 1000 population (95% CI)
2014	135 144	61.4 (61.2–61.6)	66.1 (65.8–66.5)
2015	142 537	62.2 (62.0–62.4)	68.8 (68.4–69.1)
2016	147 809	62.2 (62.1–62.5)	70.2 (69.9–70.6)
2017	154 573	63.1 (62.9–63.2)	72.1 (71.8–72.5)
2018	161 423	63.5 (63.3–63.7)	74.1 (73.7–74.4)
2019	168 690	63.9 (63.7–64.1)	76.1 (75.8–76.5)
2020	175 691	63.6 (63.5–63.8)	78.1 (77.8–78.5)
2021	186 420	64.0 (63.9–64.2)	82.6 (82.3–83.0)
2022	195 549	65.4 (65.2–65.6)	85.4 (85.1–85.8)
2023	199 040	65.7 (65.5–65.8)	84.6 (84.2–84.9)

Abbreviation: 95% CI, 95% confidence interval.

The prevalence of long‐term antidepressant use increased across all age categories during the study period (Table 3). Detailed data are presented in Supporting Information S2. The younger age groups showed higher relative increases in the prevalence of long‐term use from 2014 to 2023. The 10–24 years age group demonstrated the largest relative increase, at 110%, while the 25–39 years age group showed the second‐largest increase of 37%.

**TABLE 3:** Trends in antidepressant use patterns: 2014 versus 2023.

Age category	Year	Long‐term user %	Long‐term user % relative increase	Long‐term user prevalence (per 1000 population)	Long‐term user prevalence relative increase
10–24 years	2014	33.4 (32.8–34.1)	35%	15.4 (15.0–15.7)	
	2023	45.1 (44.6–45.6)		32.3 (31.8–32.8)	110%
25–39 years	2014	49.2 (48.8–49.7)	13%	45.0 (44.4–45.6)	
	2023	55.7 (55.3–56.0)		61.5 (60.9–62.1)	37%
40–54 years	2014	63.2 (62.8–63.5)	6%	81.8 (81.0–82.6)	
	2023	66.9 (66.5–67.2)		96.4 (95.6–97.2)	18%
55–74 years	2014	72.2 (71.8–72.5)	4%	101.8 (101.0–102.7)	
	2023	75.2 (74.9–75.5)		118.0 (117.1–118.8)	16%
75+ years	2014	73.5 (72.9–74.0)	4%	130.1 (128.4–131.8)	
	2023	76.7 (76.3–77.1)		157.8 (156.3–159.4)	21%

**FIGURE 2:**
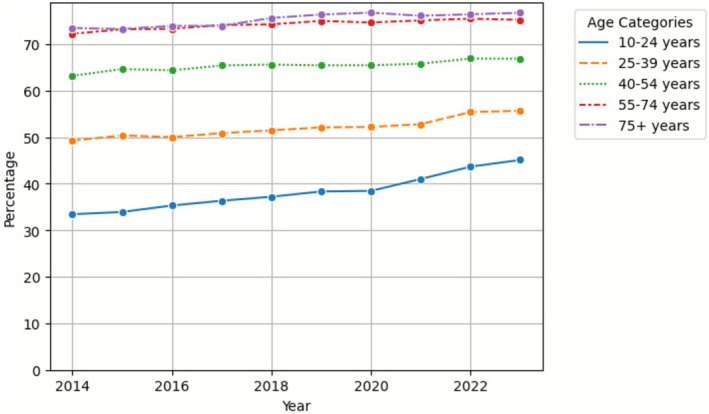
Percentage of long‐term antidepressant users among all antidepressant users by age category.

The percentage of long‐term antidepressant users relative to all antidepressant users also increased across all age categories (Figure 2). The highest percentage of long‐term users, 76.7%, was observed in the 75 years and above age group in 2023 (Table 2). However, the 10–24 year age group showed the largest relative increase in the percentage of long‐term users, rising from 33.4% in 2014 to 45.1% in 2023, an increase of 35%.

**FIGURE 3:**
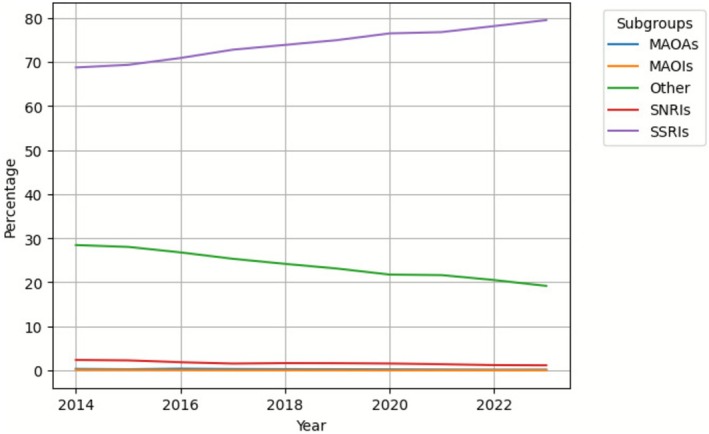
Percentage of antidepressant subcategories by ATC code (10–24 age group).

Analysis of antidepressant subcategories in the 10–24 age group showed an increased trend in the use of SSRIs throughout the study period from 68.8% (95% CI 67.5–70.0) to 79.5% (95% CI 78.9–80.1) in 2023 (Figure 3).

The average duration of antidepressant treatment episodes increased in all age categories during the study period (Table 4). Age‐stratified analysis revealed that older age groups had longer treatment durations compared to younger cohorts. However, the greatest increase in average treatment duration was observed in the 10–24 year age group, rising by 56% to reach 2.17 years in 2023.

**TABLE 4:** Average duration of treatment episode for antidepressant users by age category.

Age category	Year	DDD per day per 1000 population	Average treatment duration (years)	Relative increase in average treatment duration (2023 vs. 2014)
10–24 years	2014	31.6	1.4	
	2023	57.8	2.2	56%
25–39 years	2014	78.6	2.4	
	2023	98.4	3.0	26%
40–54 years	2014	127.6	3.9	
	2023	146.7	4.7	21%
55–74 years	2014	139.4	5.1	
	2023	164.5	6.5	27%
75+ years	2014	137.7	3.7	
	2023	170.4	4.8	29%
All ages	2014	97.7	3.3	
	2023	122.1	4.1	25%

**FIGURE 4:**
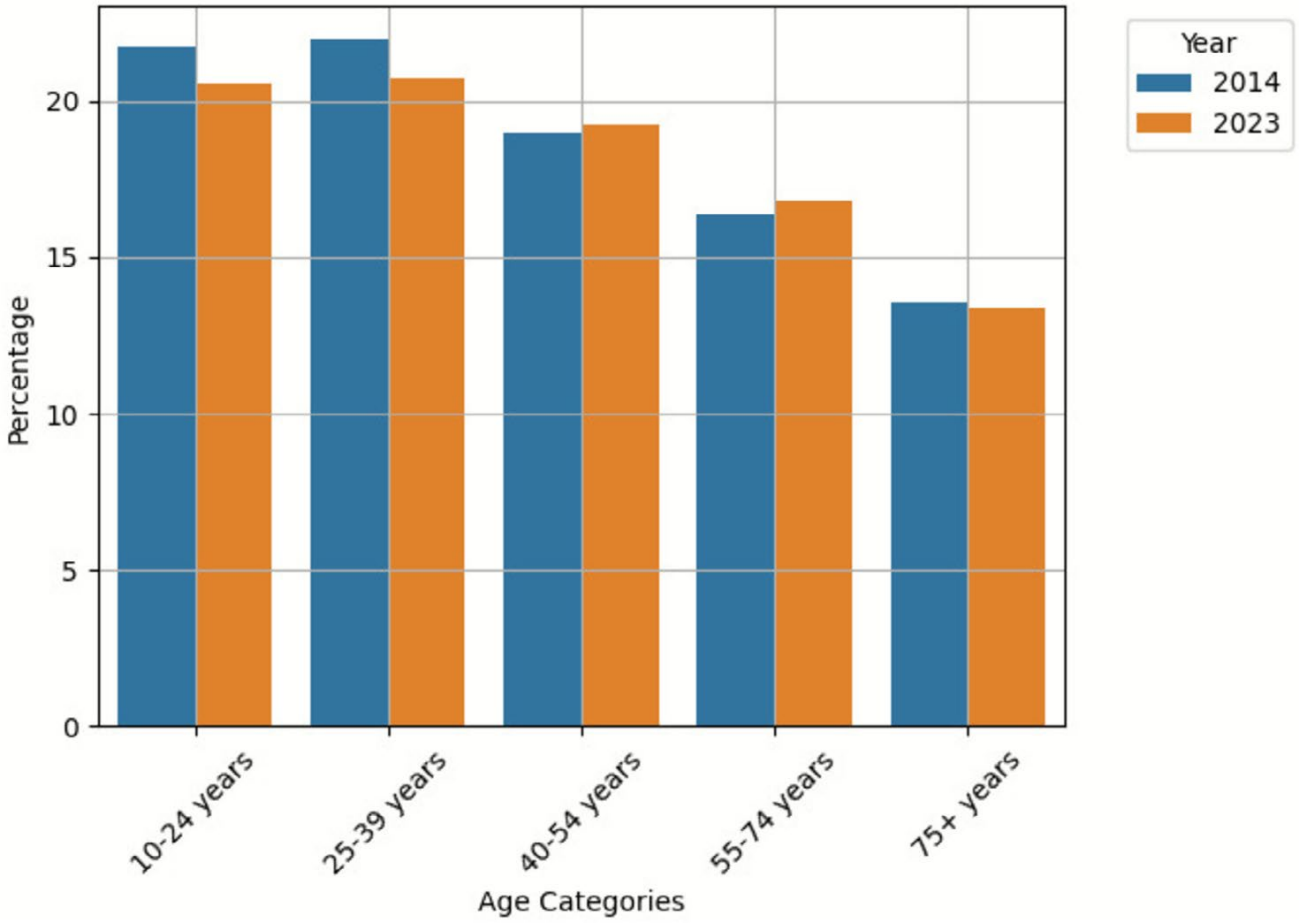
Percentage of long‐term antidepressant users with apparent dose reduction by age group, 2014 versus 2023.

We analyzed the annual percentage of long‐term antidepressant users who experienced apparent dose reduction attempts. Overall, the percentage of users with apparent dose reduction attempts remained relatively stable between 2014 and 2023, at 17.90% and 17.83%, respectively. When stratified by age category, only the 40–54 and 55–74 years age groups showed slight increases, while other age categories demonstrated slight decreases in 2023 compared to 2014 (Figure 4).

## 4 Discussion

This retrospective analysis of the PBS 10% dataset reveals significant temporal trends in long‐term antidepressant utilisation in Australia from 2014 to 2023. Our findings demonstrate an increase in overall antidepressant prevalence, accompanied by an increased proportion of long‐term users among the total antidepressant users. Females maintained a higher prevalence of long‐term antidepressant use than males throughout the study period. Age‐stratified analysis showed the most significant rise in long‐term antidepressant use in the 10–24‐year age group. The duration of average treatment episodes increased across all age cohorts as well.

During the past decade, there were no major guideline changes regarding long‐term antidepressant management in Australia. The Royal Australian and New Zealand College of Psychiatrists (RANZCP) Clinical Practice Guidelines published in 2015, 2017 and 2021 consistently recommended continuation of treatment for 6–12 months after symptom remission, with extended maintenance treatment reserved for recurrent depression [1, 27]. Despite this, our analysis revealed a substantial increase in the prevalence of long‐term use. This trend raises important questions about potential over‐prescription, difficulty in withdrawing from therapy and suboptimal deprescribing practices. Healthcare providers might continue treatment through a cautious approach, driven by concerns about potential withdrawal effects and a reluctance to deprescribe, fearing destabilization of well‐settled patients, even when clear clinical indications for continued use may be absent [28, 29].

We aimed to identify potential trends in deprescribing practices by analysing changes in the proportion of long‐term users among all antidepressant users, changes in the mean duration of treatment episodes, and the percentage of users with antidepressant apparent dose reductions during the study period. Our analysis revealed an increasing trend in the proportion of long‐term antidepressant users across all age categories. Furthermore, the mean treatment duration also increased across all age groups during this period. There was no significant change in the percentage of long‐term users experiencing a DDD reduction, which remained relatively stable. These findings of a higher proportion of long‐term users, lengthened treatment episode durations and no increase in antidepressant apparent dose reduction patterns suggest suboptimal deprescribing practices. This finding highlights the need for enhanced deprescribing strategies in antidepressant management, necessitating a comprehensive and innovative approach to identify patients suitable for deprescribing. While deprescribing guidelines and tapering plans have been developed [30], technological and analytical approaches, such as predictive modelling and AI‐based algorithms, may assist in enabling more precise patient stratification to define patients' risk levels [31, 32] and help identify low‐risk patients for medication withdrawal by analysing their clinical variables.

Analysis of the 10–24 age group revealed concerning trends. Compared to 2014, the 10–24 age group showed an increase in the incidence proportion, and the prevalence of long‐term antidepressant use in this age group rose by 110% during this study period. In addition, the average treatment duration of antidepressants for the 10–24 age group increased by 56% in 2023 compared to 2014, marking the highest increase among all age categories. Multiple contextual factors may contribute to this observed trend and warrant a comprehensive investigation in future research. The reduction of social stigma surrounding mental health has potentially encouraged young people to seek medical intervention more proactively [33]. Social media has become increasingly prevalent in the last decade, and multiple studies have documented its significant correlation with mental health disorders in younger populations, with intensive social media usage potentially contributing to increased psychological distress [34, 35, 36]. The COVID‐19 pandemic was associated with the exacerbation of psychological distress, correlating with increased antidepressant dispensing rates [37]. The expansion of healthcare services, particularly telehealth platforms, has likely facilitated improved access to continuous mental health services for young populations [38]. However, the unnecessary initiation of pharmacological interventions, suboptimal deprescribing practices and the overprescription of medications in this young age group may also be contributing to our observed results. According to the Australian and New Zealand College of Psychiatrists' Clinical Practice Guidelines for Mood Disorders, pharmacological management should not be considered the primary treatment modality for depression in children and adolescents [1]. Instead, the guidelines recommend psychological approaches, particularly cognitive behavioral therapy (CBT), even in cases of moderate to severe depression in this population.

The marked increase in mean treatment duration and the reduction in the percentage of apparent dose reductions suggest a shift toward prolonged medication use. Once antidepressant treatment is initiated, patients may continue medication use even in the absence of clinical indications. Therefore, instances of unnecessary pharmacological treatment initiation for depression in children and adolescents require thorough evaluation, as early inappropriate prescribing can lead to extended, clinically unwarranted medication exposure.

Antidepressants were the most commonly dispensed mental‐health‐related medicines in Australia in 2022–2023 [39]. Our results show that the growth in antidepressant prescriptions is predominantly driven by extended duration of use rather than an increase in incident cases, aligning with previous similar studies [9, 40]. It is crucial to evaluate whether the underutilisation of non‐pharmacological interventions contributes to the observed trend in long‐term antidepressant use. Existing research has identified significant barriers to the wider implementation of non‐pharmacological interventions in mental health, including inadequate funding, limited human resources, negative attitudinal barriers and insufficient awareness [41]. These systemic challenges highlight the need for comprehensive research investigating the complex relationships between non‐pharmacological management practices, healthcare provider attitudes and patient acceptance.

## 5 Limitations

This study has several limitations related to the data source and methodological approach. In defining long‐term medication use, we applied a criterion of continuous use that allowed up to a 60‐day gap between successive medication supplies. We also assumed that the selected medications were primarily prescribed for the management of mood disorders. Since dosage information was not available in the data source, our ‘apparent dose reduction’ measure cannot definitively distinguish dose changes from supply duration changes, and likely overestimates true intentional dose reduction attempts. Treatment duration estimates were calculated using the prevalence‐incidence ratio method rather than actual supply time periods due to left and right censoring issues in our study period, which may introduce bias compared to actual duration calculations.

## 6 Conclusion

The study reveals increasing trends in long‐term antidepressant use across all age groups during the study period. The notably high increase in the prevalence of long‐term use in the 10–24 age group is a particularly concerning finding, highlighting the necessity for future research to comprehensively understand the underlying mechanisms driving prolonged antidepressant use in younger populations. The continued increase in treatment episode duration and minimal increases in apparent dose reduction attempts across all age groups raise questions about potential overprescription and the insufficient implementation of deprescription strategies, and the limited utilisation of non‐pharmacological interventions. A comprehensive approach to mental health management is crucial, focusing on the development of effective deprescription strategies and the integration of social, psychological and innovative technological solutions.

### 6.1 Plain Language Summary

Antidepressants are commonly used to treat depression and mood disorders. They are usually recommended for short‐term use, around 6–12 months, unless a person has ongoing or repeated symptoms. However, many people are prescribed these medicines for much longer, which can increase the risk of side effects and make stopping more difficult due to withdrawal symptoms. This study looked at how the long‐term use of antidepressants has changed in Australia from 2014 to 2023 using national medicine dispensing data. It found that long‐term use increased steadily, with more people taking antidepressants for over a year. The biggest rise was seen in the 10–24 age group, where long‐term use more than doubled. The average time people stayed on antidepressants also increased, especially among younger users. There was no clear change in the percentage of people who reduced their antidepressant strength during this time. The sharp increase among younger users is a concerning finding. It highlights the need for further research to understand whether this is due to overuse, prescribing practices, or other factors. The study also points to the need for better strategies and tools to support safe and effective deprescribing.

## Ethics Statement

Approval was obtained from the Department of Human Services External Request Evaluation Committee (EREC) No: RMS3693.

## Conflicts of Interest

The authors declare no conflicts of interest.

## Supporting information


**Supporting Information: S1.** Sensitivity analysis.
**Table S1:** Antidepressant user distribution from 2014 to 2023.
**Figure S1:** Long‐term antidepressant use prevalence (2014–2023) by supply gap thresholds (15, 30 and 60 days).
**Table S2:** Long‐term antidepressant user distribution—supply days gap ≤ 15 days.
**Table S3:** Long‐term antidepressant user distribution—supply days gap ≤ 30 days.
**Table S4:** Long‐term antidepressant user distribution—supply days gap ≤ 60 days.

**Supporting Information: S2.** Trends in antidepressant use patterns by age category (2014 vs. 2023).
