# Supplementary material for: Increasing Prevalence of Long‐Term Antidepressant Use in Australia: A Retrospective Observational Study
Source: Pharmacoepidemiol Drug Saf. 2025 Nov 15;34(11):e70267. doi: 10.1002/pds.70267 (PMC12619122; doi:10.1002/pds.70267)
Supplement: Supplementary file 1 — Supporting Information: S1. Sensitivity analysis. Table S1: Antidepressant user distribution from 2014 to 2023. Figure S1: Long‐term antidepressant use prevalence (2014–2023) by supply gap thresholds (15, 30 and 60 days). Table S2: Long‐term antidepressant user distribution—supply days gap ≤ 15 days. Table S3: Long‐term antidepressant user distribution—supply days gap ≤ 30 days. Table S4: Long‐term antidepressant user distribution—supply days gap ≤ 60 days. [file PDS-34-e70267-s001.docx]

## Supplementary Material 1 : Sensitivity analysis

To determine an appropriate interval between prescription supply dates for defining continuous use, we conducted a sensitivity analysis. We varied the maximum gap interval between supplies from 15, 30 and 60 days.

Table 1: Antidepressant user distribution from 2014-2023

| Year | Incident Antidepressant users | Prevelant Antidepressant users | Incident users (%) | Incident rate *per 1,000 population* | Antidepressant users’ prevalence *per 1,000 population* |
| --- | --- | --- | --- | --- | --- |
| 2014 | 60124 | 220201 | 27.3 | 29.4 (29.2-29.6) | 107.7 (107.3-108.2) |
| 2015 | 61441 | 229009 | 26.83 | 29.6 (29.4-29.9) | 110.5 (110.1-110.9) |
| 2016 | 62465 | 237432 | 26.31 | 29.7 (29.4-29.9) | 112.8 (112.4-113.2) |
| 2017 | 63452 | 245127 | 25.89 | 29.6 (29.4-29.8) | 114.4 (114.0-114.8) |
| 2018 | 64963 | 254200 | 25.56 | 29.8 (29.6-30.0) | 116.6 (116.2-117.1) |
| 2019 | 66644 | 264029 | 25.24 | 30.1 (29.8-30.3) | 119.1 (118.7-119.5) |
| 2020 | 70050 | 276092 | 25.37 | 31.1 (30.9-31.4) | 122.7 (122.3-123.2) |
| 2021 | 73045 | 291106 | 25.09 | 32.4 (32.1-32.6) | 129.0 (128.6-129.5) |
| 2022 | 70122 | 298959 | 23.46 | 30.6 (30.4-30.9) | 130.6 (130.2-131.1) |
| 2023 | 69251 | 303104 | 22.85 | 29.4 (29.2-29.6) | 128.8 (128.4-129.2) |


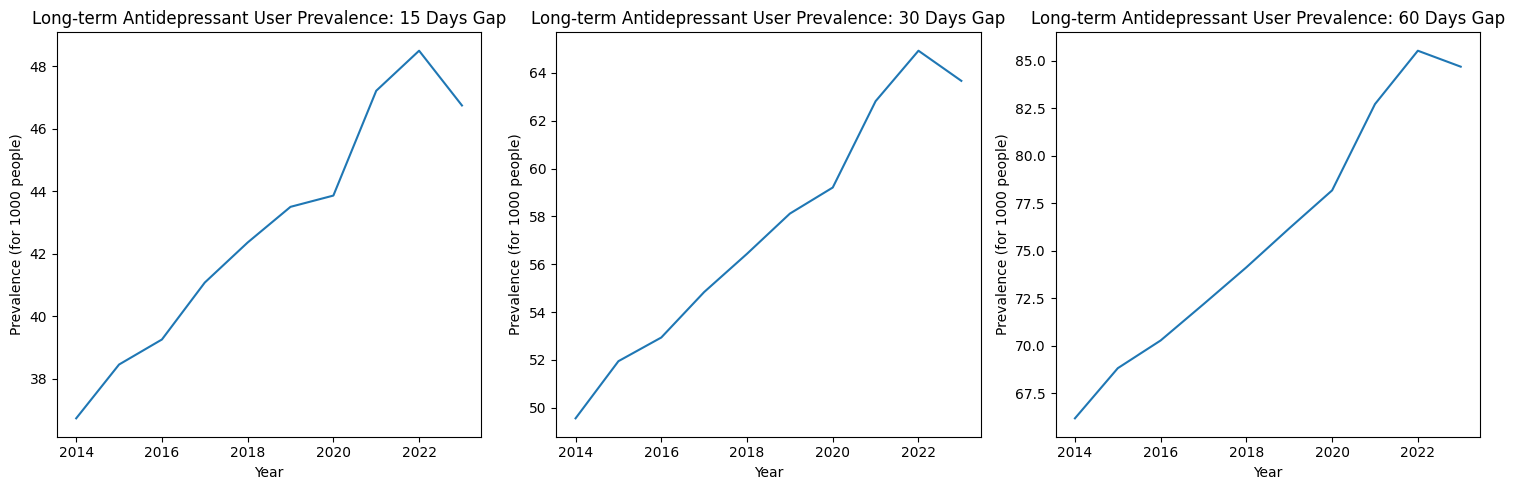


Figure 1 : Long-Term Antidepressant Use Prevalence (2014–2023) by Supply Gap Thresholds (15, 30, 60 Days)

Table 2: : Long-term Antidepressant User Distribution - Supply Days Gap ≤15 Days

| Year | Long term Antidepressant users | Long-term users % among all users | Long-term users’ prevalence *per 1,000 population* |
| --- | --- | --- | --- |
| 2014 | 75090 | 34.10 | 36.7 (36.5-37.0) |
| 2015 | 79725 | 34.81 | 38.5 (38.2-38.7) |
| 2016 | 82644 | 34.81 | 39.3 (39.0-39.5) |
| 2017 | 88032 | 35.91 | 41.1 (40.8-41.3) |
| 2018 | 92329 | 36.32 | 42.4 (42.1-42.6) |
| 2019 | 96423 | 36.52 | 43.5 (43.2-43.8) |
| 2020 | 98668 | 35.74 | 43.9 (43.6-44.1) |
| 2021 | 106500 | 36.58 | 47.2 (46.9-47.5) |
| 2022 | 110980 | 37.12 | 48.5 (48.2-48.8) |
| 2023 | 109992 | 36.29 | 46.7 (46.5-47.0) |

Table 3: : Long-term Antidepressant User Distribution - Supply Days Gap ≤30 Days

| Year | Long term Antidepressant users | Long-term users % among all users | Long-term users’ prevalence *per 1,000 population* |
| --- | --- | --- | --- |
| 2014 | 101202 | 45.96 | 49.5 (49.2-49.8) |
| 2015 | 107587 | 46.98 | 51.9 (51.6-52.2) |
| 2016 | 111333 | 46.89 | 52.9 (52.6-53.2) |
| 2017 | 117380 | 47.89 | 54.8 (54.5-55.1) |
| 2018 | 122872 | 48.34 | 56.4 (56.1-56.7) |
| 2019 | 128671 | 48.73 | 58.0 (57.8-58.4) |
| 2020 | 133010 | 48.18 | 59.1 (58.8-59.4) |
| 2021 | 141521 | 48.61 | 62.7 (62.4-63.0) |
| 2022 | 148384 | 49.63 | 64.8 (64.5-65.2) |
| 2023 | 149579 | 49.35 | 63.6 (63.2-63.9) |

Table 4: : Long-term Antidepressant User Distribution - Supply Days Gap ≤60 Days

| Year | Long term Antidepressant users | Long-term users % among all users | Long-term users’ prevalence *per 1,000 population* |
| --- | --- | --- | --- |
| 2014 | 135144 | 61.37 | 66.1 (65.8-66.5) |
| 2015 | 142537 | 62.24 | 68.8 (68.4-69.1) |
| 2016 | 147809 | 62.25 | 70.2 (69.9-70.6) |
| 2017 | 154573 | 63.06 | 72.1 (71.8-72.5) |
| 2018 | 161423 | 63.5 | 74.1 (73.7-74.4) |
| 2019 | 168690 | 63.89 | 76.1 (75.8-76.5) |
| 2020 | 175691 | 63.63 | 78.1 (77.8-78.5) |
| 2021 | 186420 | 64.04 | 82.6 (82.3-83.0) |
| 2022 | 195549 | 65.41 | 85.4 (85.1-85.8) |
| 2023 | 199040 | 65.67 | 84.6 (84.2-84.9) |
