# Supplementary material for: Increasing Prevalence of Long‐Term Antidepressant Use in Australia: A Retrospective Observational Study
Source: Pharmacoepidemiol Drug Saf. 2025 Nov 15;34(11):e70267. doi: 10.1002/pds.70267 (PMC12619122; doi:10.1002/pds.70267)
Supplement: Supplementary file 2 — Supporting Information: S2. Trends in antidepressant use patterns by age category (2014 vs. 2023). [file PDS-34-e70267-s002.docx]

## Supplementary Material 2: Trends in antidepressant use patterns by Age category ( 2014 versus 2023)

| Age category | Year | New users | Prevalent users | Long-term users | New user % | Long- term user % | Long-term user % relative increase | Antidepressant incident rate (per 1,000 population) | Antidepressant user prevalence (per 1,000 population) | Long-term user prevalence (per 1,000 population) | Long-term user prevalence relative increase |  |
| --- | --- | --- | --- | --- | --- | --- | --- | --- | --- | --- | --- | --- |
|  |  |  |  |  |  |  |  |  |  |  |  |  |
| 10-24 years | 2014 | 10278 | 20846 | 6968 | 49.3 (48.6-50.0) | 33.4 (32.8-34.1) | 35% | 22.7 (22.2-23.1) | 46.0 (45.4-46.6) | 15.4 (15.0-15.7) |  |  |
|  | 2023 | 13292 | 35797 | 16143 | 37.1 (36.6-37.6) | 45.1 (44.6-45.6) |  | 26.6 (26.2-27.1) | 71.7 (70.9-72.4) | 32.3 (31.8-32.8) | 110% |  |
| 25-39 years | 2014 | 16278 | 45843 | 22575 | 35.5 (35.1-36.0) | 49.2 (48.8-49.7) | 13% | 32.5 (32.0-32.9) | 91.4 (90.6-92.2) | 45.0 (44.4-45.6) |  |  |
|  | 2023 | 18951 | 64684 | 36000 | 29.3 (28.9-29.6) | 55.7 (55.3-56.0) |  | 32.4 (31.9-32.8) | 110.5 (109.7-111.3) | 61.5 (60.9-62.1) | 37% |  |
| 40-54 years | 2014 | 15498 | 61714 | 38986 | 25.1 (24.8-25.4) | 63.2 (62.8-63.5) | 6% | 32.5 (32.0-33.0) | 129.5 (128.6-130.5) | 81.8 (81.0-82.6) |  |  |
|  | 2023 | 15759 | 73205 | 48943 | 21.5 (21.2-21.8) | 66.9 (66.5-67.2) |  | 31.0 (30.6-31.5) | 144.1 (143.2-145.1) | 96.4 (95.6-97.2) | 18% |  |
| 55-74 years | 2014 | 12520 | 65062 | 46974 | 19.2 (18.9-19.6) | 72.2 (71.8-72.5) | 4% | 27.1 (26.7-27.6) | 141.0 (140.0-142.0) | 101.8 (101.0-102.7) |  |  |
|  | 2023 | 13921 | 86743 | 65218 | 16.1 (15.8-16.3) | 75.2 (74.9-75.5) |  | 25.2 (24.8-25.6) | 156.9 (155.9-157.8) | 118.0 (117.1-118.8) | 16% |  |
| 75+ years | 2014 | 5550 | 26736 | 19641 | 20.8 (20.3-21.2) | 73.5 (72.9-74.0) | 4% | 36.8 (35.8-37.7) | 177.1 (175.2-179.0) | 130.1 (128.4-131.8) |  |  |
|  | 2023 | 7328 | 42675 | 32736 | 17.2 (16.8-17.5) | 76.7 (76.3-77.1) |  | 35.3 (34.5-36.1) | 205.8 (204.0-207.5) | 157.8 (156.3-159.4) | 21% |  |
